# Supplementary material for: Synthesis of Mesoporous Tetragonal ZrO2, TiO2 and Solid Solutions and Effect of Colloidal Silica on Porosity
Source: Molecules. 2024 Jul 11;29(14):3278. doi: 10.3390/molecules29143278 (PMC11279741; doi:10.3390/molecules29143278)
Supplement: Supplementary file 1 [file molecules-29-03278-s001.zip › molecules-3089112-supplementary.pdf]

Supplementary Materials

Synthesis of Mesoporous Tetragonal ZrO_2, TiO_2 and Solid Solutions and Effect of Colloidal Silica on Porosity

Linggen Kong * , Inna Karatchevtseva, Tao Wei and Jessica Veliscek-Carolan

Australian Nuclear Science and Technology Organisation, New Illawarra Road, Lucas Heights, NSW 2234, Australia; ikm@ansto.gov.au (I.K.); tao@ansto.gov.au (T.W.); jvc@ansto.gov.au (J.V.-C.)
* Correspondence: lnk@ansto.gov.au

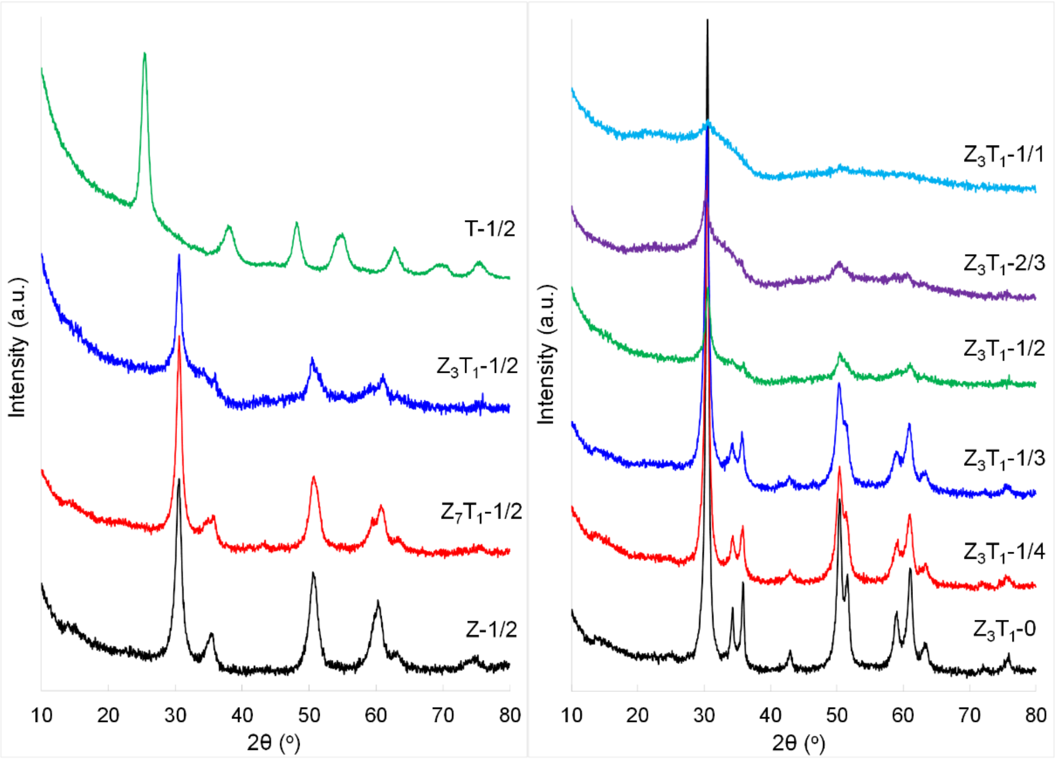

Figure S1. XRD patterns of the calcined powders prior to leaching silica.

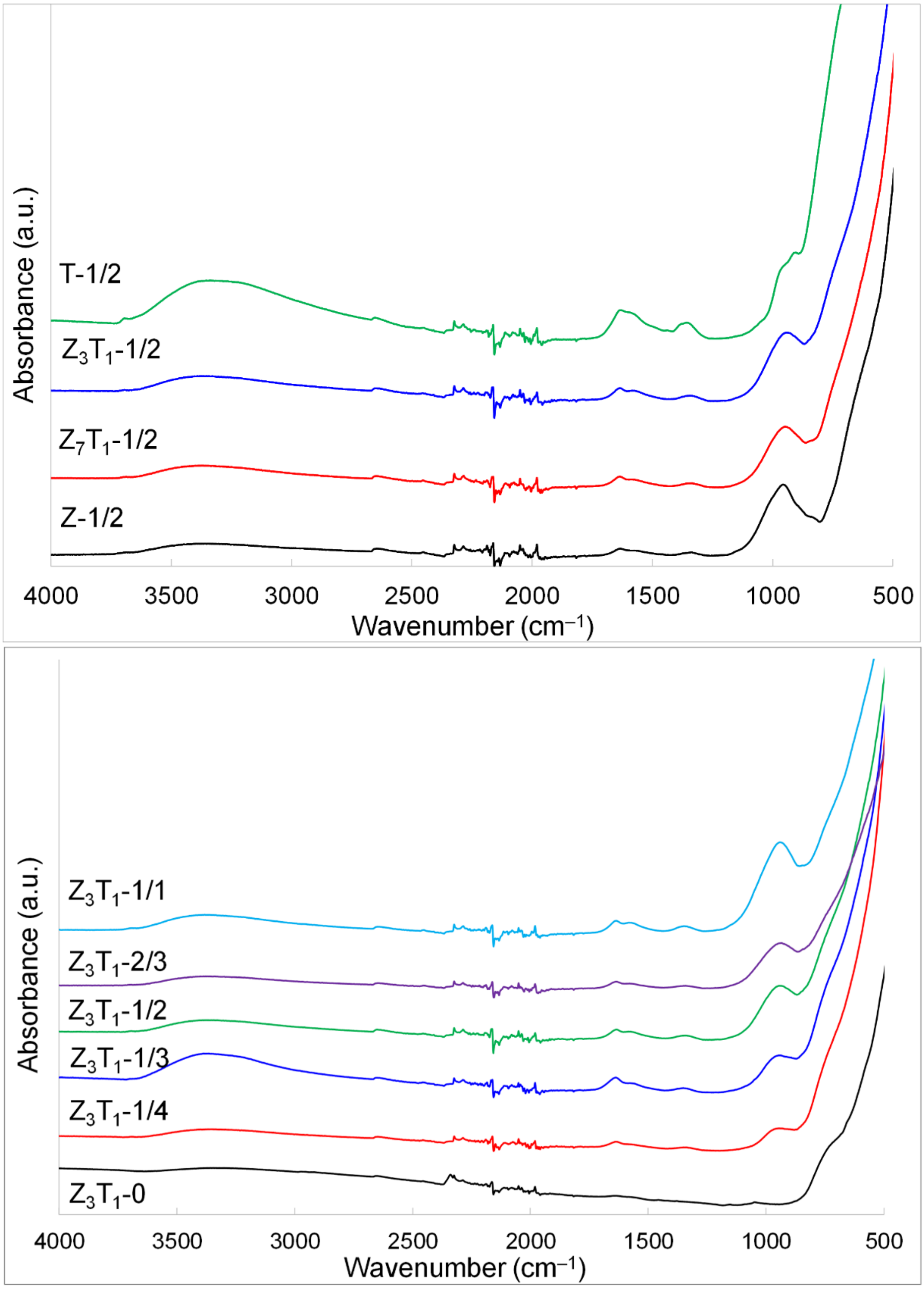

Figure S2. FTIR spectra of the silica leached powders with broad wavenumber range.

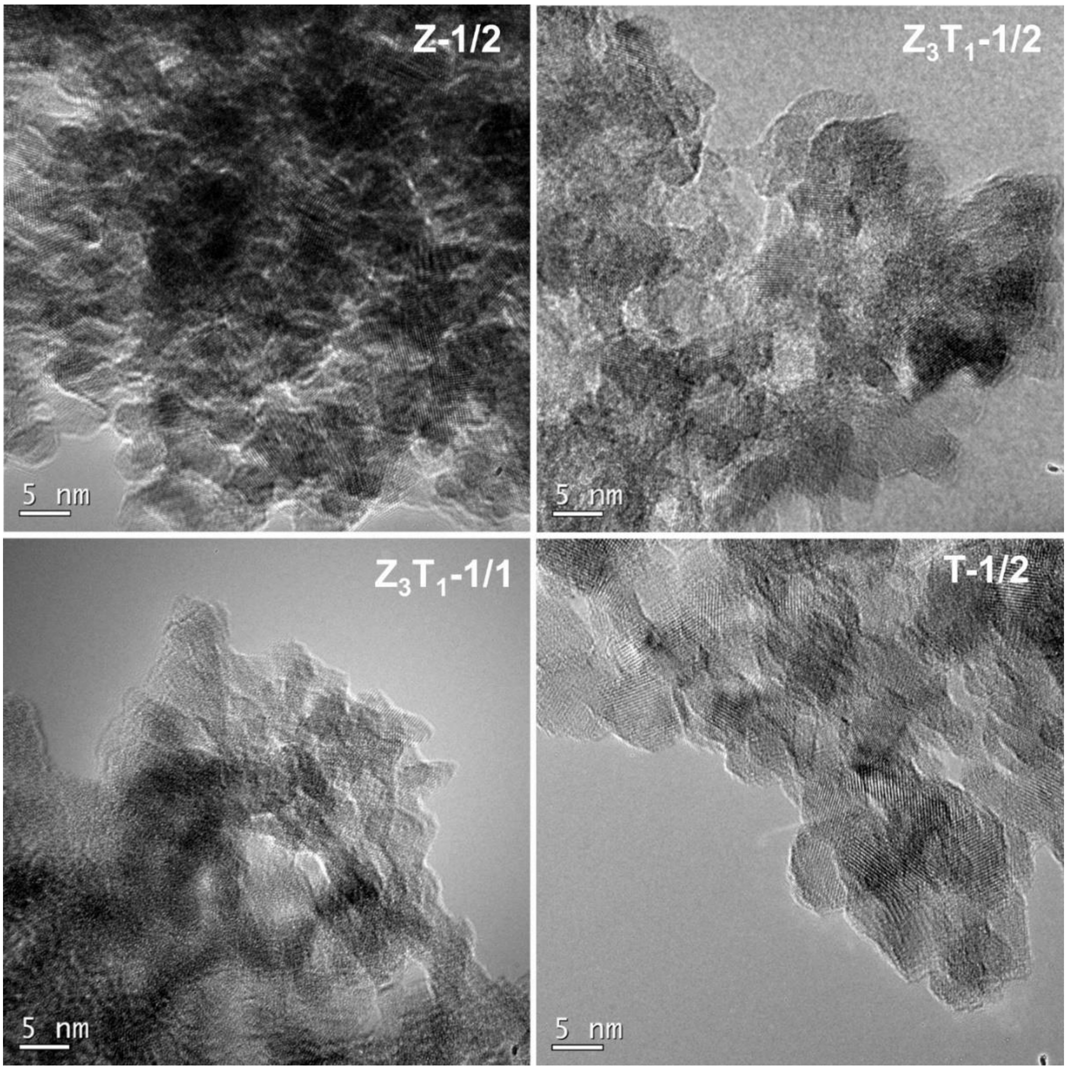

Figure S3. TEM images of the silica leached powders with high magnification.

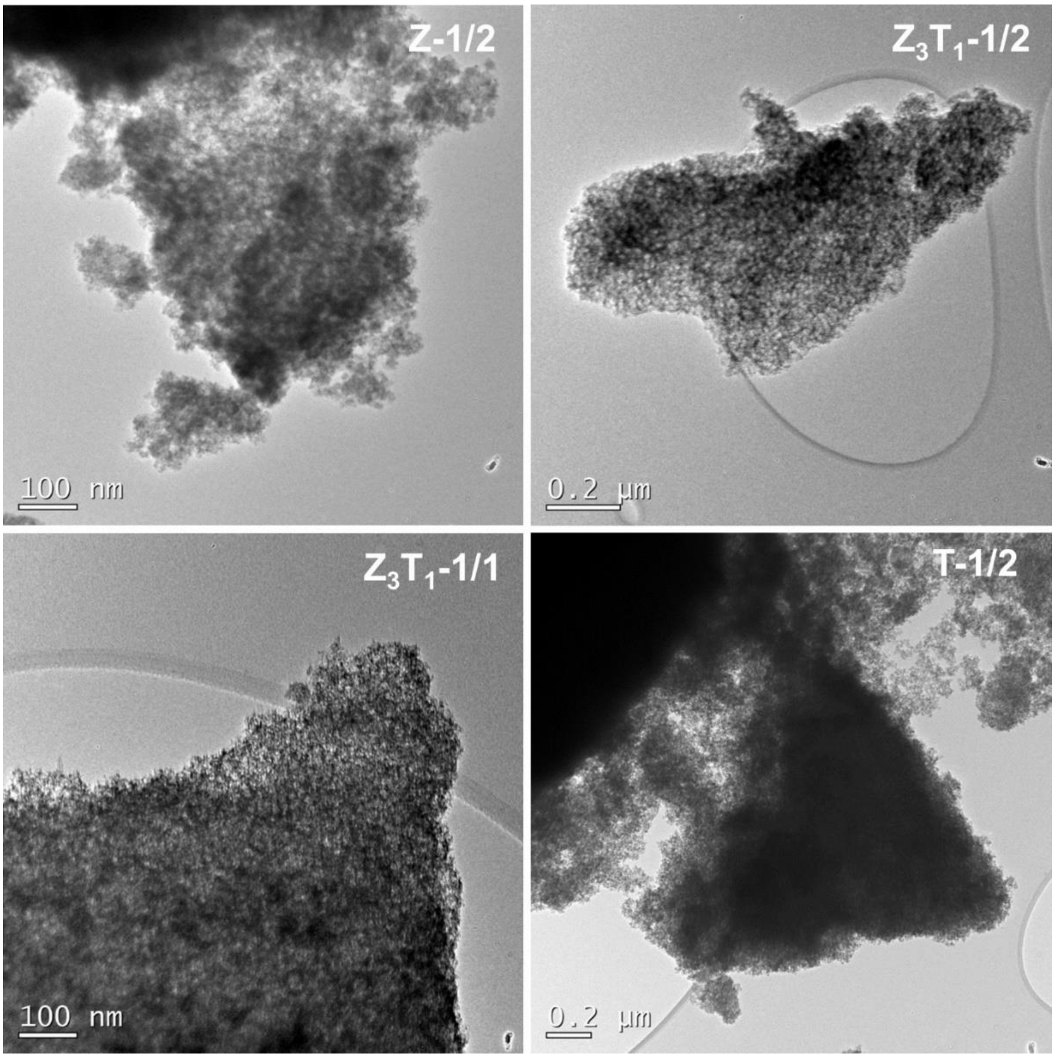

Figure S4. TEM images of the silica leached powders with low magnification.

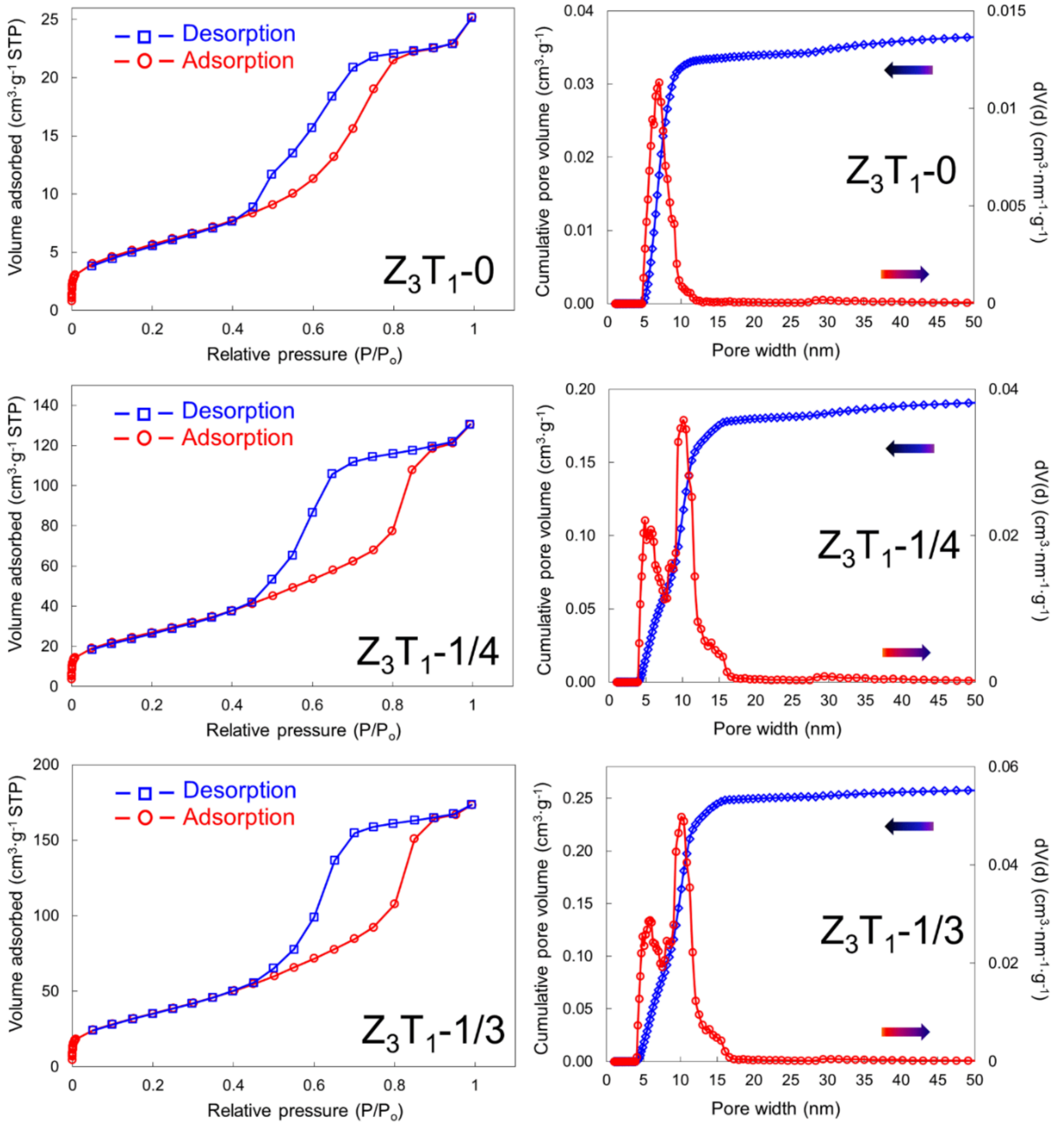
Desorption
Adsorption
Z3T1-0
Volume adsorbed (cm3·g-1 STP)
Relative pressure (P/Po)
Cumulative pore volume (cm3·g-1)
dV(d) (cm3·nm-1·g-1)
Pore width (nm)
Z3T1-1/4
Z3T1-1/3

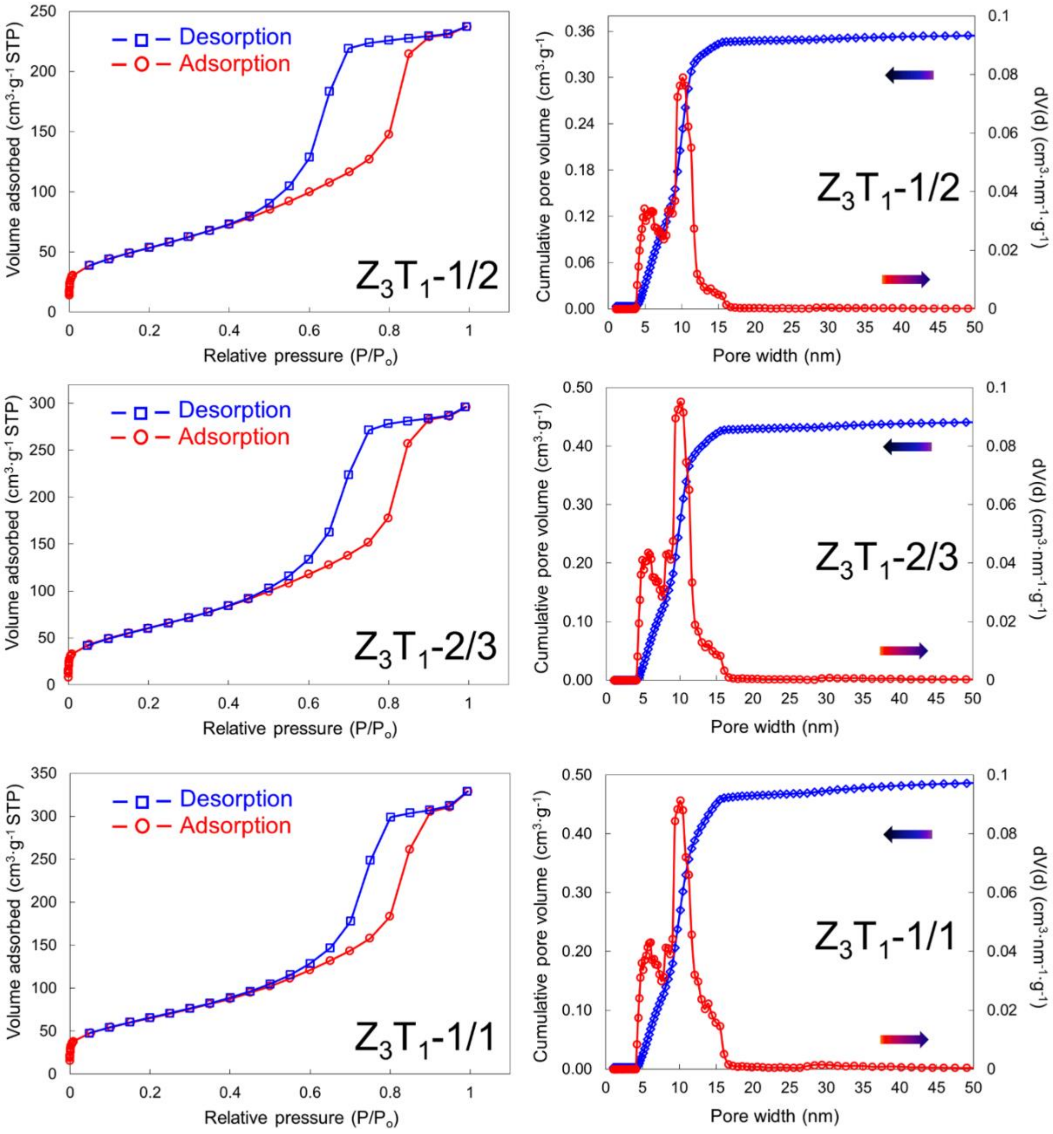

Figure S5. Nitrogen sorption isotherms, pore size distributions and the corresponding cumulative pore volumes based on DFT analysis of the silica leached $Zr_{0.75}Ti_{0.25}O_2$ powders, at various silica to $Zr_{0.75}Ti_{0.25}O_2$ weight ratios. Sample descriptions are shown in Table 1.

Table S1. Surface area estimation of the $Zr_{0.75}Ti_{0.25}O_2$ powders at various silica to $Zr_{0.75}Ti_{0.25}O_2$ weight ratios.

Sample	Z_3T_1-0	Z_3T_1-1/4	$Z_3T_1$1-1/3	Z_3T_1-1/2	Z_3T_1-2/3	Z_3T_1-1/1*
SiO_2/ZT (*w*/*w*)	0	1:4	1:3	1:2	2:3	1:1
ZT $\Phi_{particle}$ (nm)	21.2	12.31	11.23	10.05	4.76	1.43
ZT mass (g)	1	1	1	1	1	1
ZT ϱ ($g{\cdot}cm^{-3}$)	5.638	5.638	5.638	5.638	5.638	5.638
ZT V_{Tot} (cm^3)	0.177	0.177	0.177	0.177	0.177	0.177
ZT $V_{particle}$ (cm^3)	4.99×10^{-18}	9.77×10^{-19}	7.42×10^{-19}	5.32×10^{-19}	5.65×10^{-20}	1.53×10^{-21}
ZT N_{Tot} (g^{-1})	3.56×10^{16}	1.82×10^{17}	2.39×10^{17}	3.34×10^{17}	3.14×10^{18}	1.16×10^{20}
ZT $A_{particle}$ (nm^2)	1.41×10^3	4.76×10^2	3.96×10^2	3.17×10^2	7.12×10	6.42
ZT A_{Cal} ($m^2{\cdot}g^{-1}$)	50.2	86.5	94.8	106	224	744
ZT A_{Exp} ($m^2{\cdot}g^{-1}$)	19.4	89.3	121	187	204	226
Silica mass (g)	0	0.25	0.333	0.5	0.667	1
Silica A_{Cal} ($m^2{\cdot}g^{-1}$)		55	73.3	110	147	220
$A_{Cal\text{-}silica}/A_{Cal\text{-}ZT}$		0.64	0.77	1.04	0.66	0.30
$A_{Cal\text{-}silica}/A_{Exp\text{-}ZT}$		0.62	0.61	0.59	0.72	0.97

ZT: $Zr_{0.75}Ti_{0.25}O_2$ materials. $\Phi_{particle}$: average diameter of ZT particle, assuming the crystallite is spherical and crystallite size equals to the particle diameter. ϱ: density of $Zr_{0.75}Ti_{0.25}O_2$, V_{Tot}: total volume of ZT particles, $V_{particle}$: volume per ZT particle, N_{Tot}: total number of ZT particles, $A_{particle}$: surface area per ZT particle, A_{cal}: calculated surface area, A_{Exp}: Experimental surface area by DFT modelling, *: low confidence figures for this sample.

Molecular weight and cell volume of $Zr_{0.75}Ti_{0.25}O_2$ are 112.383 g mol^{-1} and 66.1981 $Å^3$ [S1], respectively. So the calculated theoretical density of this material is 5.638 g cm^{-3}.

For 1 g of the crystalline ZT, the total volume of ZT particles (V_{Tot}) can be obtained using density ϱ being 5.638 g cm^{-3}. It is assumed that the ZT crystallite is spherical and crystallite size equals to the particle diameter, so both the volume per ZT particle ($V_{particle}$) and the surface area per ZT particle ($A_{particle}$) can be calculated using the crystallite size in Table 1. The total number of ZT particles (N_{Tot}) equals to $V_{Tot}/V_{particle}$. The calculated surface area of the ZT material (A_{cal}) is $A_{particle}$ x N_{Tot}, and the experimental surface area of the ZT material (A_{Exp}) is the nitrogen adsorption analysis results by DFT modelling (Table 4). Amorphous colloidal silica (Ludox® HS-30) has surface area ~220 m^2 g^{-1}. Based on the silica to ZT weight ratio, the silica mass and then the calculated surface area of the silica ($A_{Cal\text{-}silica}$) are obtained. As a result, both the silica to calculated ZT surface area ratio and the silica to experimental ZT surface area ratio can be estimated.

Reference

S1 Kong, L.; Karatchevtseva, I.; Zhu, H.; Qin, M.J.; Aly, Z. Synthesis and microstructure characterization of tetragonal $Zr_{1-x}Ti_xO_2$ (x = 0–1) solid solutions. *J. Mater. Sci. Technol.* **2019**, *35*, 1966–1976.
